# *KLF6* depletion promotes NF-κB signaling in glioblastoma

**DOI:** 10.1038/onc.2016.507

**Published:** 2017-02-06

**Authors:** A P Masilamani, R Ferrarese, E Kling, N K Thudi, H Kim, D M Scholtens, F Dai, M Hadler, T Unterkircher, L Platania, A Weyerbrock, M Prinz, G Y Gillespie, G R Harsh IV, M Bredel, M S Carro

**Affiliations:** 1 Department of Neurosurgery, Medical Center, University of Freiburg, Freiburg, Germany; 2 Faculty of Medicine, University of Freiburg, Freiburg, Germany; 3 Department of Radiation Oncology, UAB Comprehensive Cancer Center, University of Alabama at Birmingham School of Medicine, Birmingham, AL, USA; 4 Jackson Laboratory for Genomic Medicine, Farmington, CT, USA; 5 Department of Preventive Medicine, Robert H. Lurie Comprehensive Cancer Center, Northwestern University School of Medicine, Chicago, IL, USA; 6 Institute of Neuropathology, University of Freiburg, Freiburg, Germany; 7 BIOSS Centre for Biological Signalling Studies, University of Freiburg, Freiburg, Germany; 8 Department of Neurosurgery, UAB Comprehensive Cancer Center, University of Alabama at Birmingham School of Medicine, Birmingham, AL, USA; 9 Department of Neurosurgery, Stanford University School of Medicine, Stanford, CA, USA

## Abstract

Dysregulation of the NF-κB transcription factor occurs in many cancer types. Krüppel-like family of transcription factors (KLFs) regulate the expression of genes involved in cell proliferation, differentiation and survival. Here, we report a new mechanism of NF-κB activation in glioblastoma through depletion of the *KLF6* tumor suppressor. We show that KLF6 transactivates multiple genes negatively controlling the NF-κB pathway and consequently reduces NF-κB nuclear localization and downregulates NF-κB targets. Reconstitution of KLF6 attenuates their malignant phenotype and induces neural-like differentiation and senescence, consistent with NF-κB pathway inhibition. *KLF6* is heterozygously deleted in 74.5% of the analyzed glioblastomas and predicts unfavorable patient prognosis suggesting that haploinsufficiency is a clinically relevant means of evading KLF6-dependent regulation of NF-κB. Together, our study identifies a new mechanism by which KLF6 regulates NF-κB signaling, and how this mechanism is circumvented in glioblastoma through *KLF6* loss.

## Introduction

The NF-κB transcription factor family is oncogenic through suppression of programmed cell death, and promotion of tumor growth and invasion.^1^ In tumors, NF-κB can be activated by mutations in its own genes or in its regulating genes.^2^ In the canonical pathway, NFKBIA (IκBα)^3^ interacts and sequesters the p65/p50 NF-κB heterodimer in the cytoplasm. Upon various stimuli, NFKBIA is phosphorylated and degraded allowing translocation of NF-κB in the nucleus and transcriptional activation of NF-κB targets. Although both subunits can bind to the DNA, only p65 contains a transcriptional activation domain.^4^

Mutations and enrichment of specific single-nucleotide polymorphisms and haplotypes of *NFKBIA* in human cancer suggest a role as tumor suppressor.^5, 6, 7, 8^ Other genes negatively regulate NF-κB activation, such as the TNF α-induced protein 3 (TNFAIP3; A20), a ubiquitin-editing enzyme which downregulates NF-κB signaling when binding TNFAIP3-interacting proteins 1 and 2 (TNIP1 and TNIP2, respectively).^9^

We previously found that monoallelic deletion of *NFKBIA* occurs in about 25% of glioblastomas and convey a dismal clinical prognosis.^8^ However, aberrant constitutive activation of NF-κB occurs in most glioblastomas,^10^ suggesting additional mechanisms of NF-κB activation.

KLFs regulate expression of genes involved in signal transduction, proliferation, differentiation, cell death and oncogenesis. KLF6 is a putative tumor suppressor in prostate, colorectal, hepatocellular carcinomas and glioblastoma.^11, 12, 13, 14, 15, 16, 17^ Deletion of the chromosome region containing *KLF6* (10p15) has been reported in glioblastoma,^16^ whereas mutation analyses of the *KLF6* coding region have been controversial.^16, 18, 19, 20, 21, 22^ KLF6 has been proposed to perform its tumor suppression function by promoting G1 cell cycle arrest mainly through cyclin-dependent kinase inhibitor 1A *(CDKN1A)* promoter transactivation.^15^

The *KLF6* splice variant *sv1* is aberrantly expressed in prostate, ovarian cancer and glioblastoma.^16, 23^ Upon splicing, KLF6-sv1 lacks a nuclear localization signal; therefore, it cannot transactivate KLF6 targets and supposedly is non-functional.^24^ Nevertheless, KLF6-sv1 has been shown to promote tumor progression and metastasis in various cancers.^25, 26^

Here, we employ genome-wide scanning for transcripts co-expressed with *NFKBIA*, *TNFAIP3*, *TNIP1* and *TNIP2* to identify KLF6 as a common transactivator of NF-κB-negative regulatory genes. We demonstrate that *KLF6* is frequently inactivated in glioblastoma and propose *KLF6* deletion as a new mechanism underlying NF-κB signaling increase in this tumor type.

## Results

### NF-κB-negative regulators are co-regulated in glioblastoma

**Figure 1:**
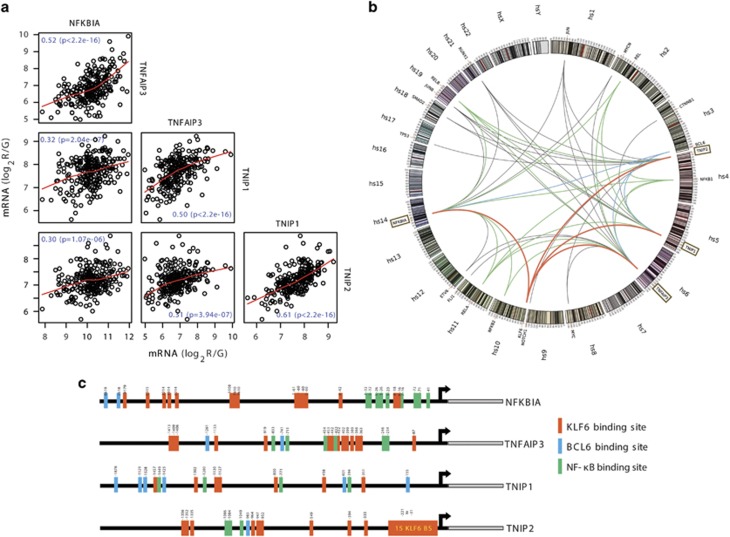
Negative regulators of NF-κB are co-regulated in glioblastoma. (**a**) Scatter plot matrix for messenger RNA expression of NF-κB control genes *NFKBIA*, *TNFAIP3*, *TNIP1* and *TNIP2* representing pairwise associations between each of these variables in 188 glioblastomas. Locally weighted least squares smooth fits indicate the appropriateness of the linear regression analyses. The corresponding *P*-values indicate the statistical significance of these relationships according to estimated slope of the regression line. (false discovery rate adjusted *q*<1 × 10^−5^). (**b**) Genome-wide correlation analysis of the four NF-κB control genes (*TNIP1*, *TNIP2*, *NFKBIA*, *TNFAIP3*) with other gene transcripts in 413 glioblastomas (significance level: *P*<1 × 10^−5^). Significant associations between the NF-κB-negative regulators and Rel-like domain-containing proteins (NFKB1, NFKB2, RELA, c-REL and RELB) are indicated by green edges. Edges connecting the control genes to KLF6 and BCL6, red and blue, respectively. (**c**) Schematic diagram of predicted binding sites of transcription factors KLF6, BCL6 and NF-κB in the promoter regions of the four NF-κB control genes (*NFKBIA*, *TNFAIP3*, *TNIP1* and *TNIP2*).

To determine whether deregulation of negative regulators of NF-κB has a role in constitutive NF-κB activation in glioblastoma, we analyzed expressions of the NF-κB regulators *NFKBIA*, *TNFAIP3*, *TNIP1* and *TNIP2* in glioblastoma patients from The Cancer Genome Atlas (TCGA). All regulators showed co-expression, suggesting a common regulation (Figure 1a). We excluded genomic co-mapping (*NFKBIA*: 14q13.2, *TNFAIP3*: 6q23.3, *TNIP1*: 5q33.1 and *TNIP2*: 4p16.3) of these genes and concurrent gene copy number variation as a cause of their co-expression (Supplementary Figure 1A). To identify common transcriptional regulators, we performed a genome-wide scan for transcripts co-expressed with the four NF-κB-negative regulators. Filtering for cancer-associated transcription factors,^27^ we identified 18 genes (Figure 1b); 5 of which encoded Rel-like domain-containing proteins forming homo- or heterodimeric NF-κB complexes and inducing NF-κB regulator genes through negative feedback regulation.^28, 29^ Another seven transcription factors were co-expressed with all the four NF-κB-negative regulators (Figure 1b), suggesting a role in their transcriptional regulation. In a complementary approach, we looked for common transcription factors binding sites by performing *in silico* promoter binding analyses (MatInspector, Genomatix, Munich, Germany) for all NF-κB-negative regulators and identified 43 transcription factors with binding sites present in all promoters (Supplementary Figure 2).

### KLF6 is a clinically relevant putative tumor suppressor

**Figure 2:**
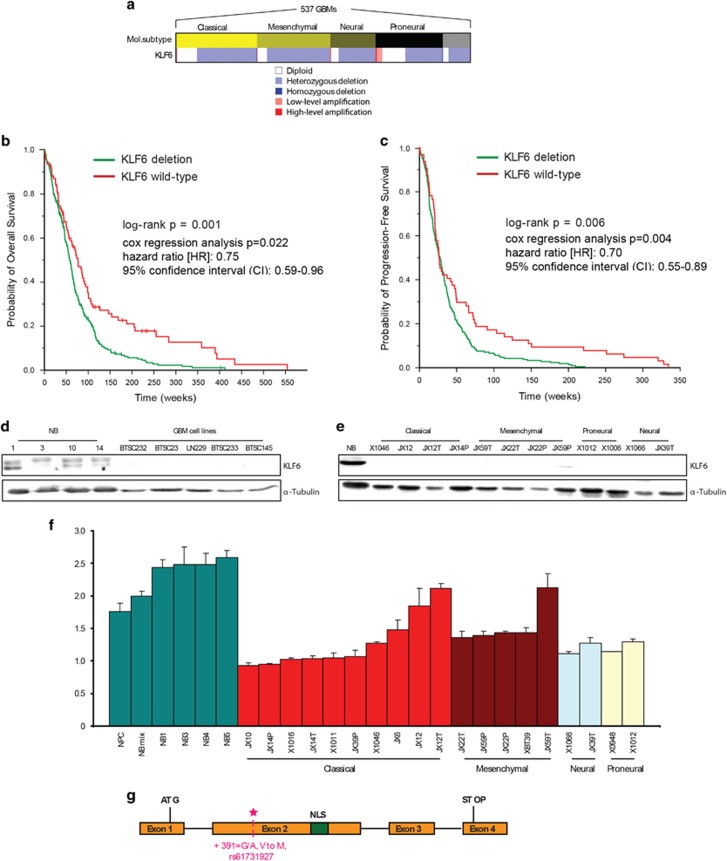
KLF6 is a clinically relevant putative tumor suppressor. (**a**) Heatmap displaying gene copy number variation analysis for *KLF6* (maps to 10p15) in 537 TCGA glioblastomas by circular binary segmentation 36 and Genomic Identification of Significant Targets in Cancer (GISTIC2). The association with four major subtypes (classical, mesenchymal, neural and proneural) of glioblastoma is shown. (**b**) Kaplan–Meier estimates of overall survival for 406 glioblastoma patients, with patients stratified into two subgroups based on whether their tumor harbored a deletion of *KLF6*. (**c**) Kaplan–Meier estimates of progression-free survival in the same patients stratified in the same manner according to *KLF6* gene status. (**d**) Immunoblotting for KLF6 in normal brain tissues, and patient-derived glioblastoma cells. (**e**) Immunoblotting for KLF6 in normal brain tissues, glioblastoma-derived xenolines. (**f**) Gene copy number analysis for KLF6 in neural progenitor cells, normal brain tissues and glioblastoma-derived xenolines. (**g**) Schematic representation of the KLF6 exon structure showing the four coding sequence exons, localization of the nuclear localization signal (NLS) in exon 2 and the new identified mutation (391 G/A, V to M).

Three transcription factors, B-cell CLL/lymphoma 6 (*BCL6*), *KLF6* and the NF-κB family, were identified in both analyses (Figure 1c). Given the expected feedback between NF-κB and NF-κB control genes, we focused on BCL6, an oncogenic repressor^30^ and KLF6, a transcriptional activator and tumor suppressor.^11^ Copy number analysis for *KLF6* (10p15) and *BCL6* (3q27) revealed heterozygous deletions of *KLF6* in 74.5% of tumors, but homozygous deletions in only 0.4% (Figure 2a). *BCL6* showed low-level amplification in 7.3% and high-level amplification in 0.7% of the tumors (data not shown). We then assessed *KLF6* and *BCL6* associations with survival in 406 patients with glioblastoma. We found no survival association for *BCL6* amplifications (log-rank *P*>0.05; data not shown), but patients with *KLF6* deletions had significantly shorter progression-free survival and overall survival than those without *KLF6* deletions (Figures 2b and c).

Consistent with the high *KLF6* heterozygous deletion frequency observed in our analysis, KLF6 expression in glioblastoma was reduced compared with normal brain samples (non-malignant cortical samples obtained from epilepsy surgery, NB) (Figure 2d). In a panel of glioblastoma-derived xenolines characterized for molecular subtype, KLF6 protein did not show association with any specific GBM subtype^31^ (Figure 2e). Consistently, *KLF6* deletion was proportionally distributed among the four subtypes (Figure 2a). Copy number analysis of the same cell panel showed frequent loss of one allele (Figure 2f). However, three xenolines (JX12, JX12T and JX59T) without *KLF6* deletion still lacked KLF6 expression hinting at additional silencing mechanisms.

Given previous contradictory results regarding *KLF6* mutations in glioblastoma, we sequenced the *KLF6* sequence upstream of the ATG (431 bp), CDS exons 1–4 and intron–exon boundaries in 45 glioblastoma samples. We identified several previously reported single-nucleotide polymorphisms (data not shown) and a new mutation (391 G/A, V to M) in one patient (frequency: 0.02) (Figure 2g), confirming that *KLF6* is rarely mutated in glioblastoma.^22^

### KLF6 transactivates NF-κB control genes in glioblastoma

**Figure 3:**
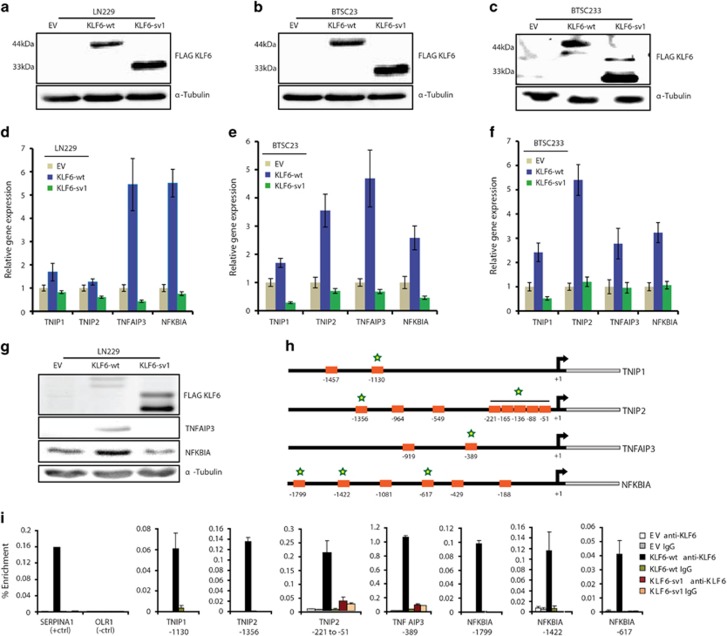
KLF6 induces NF-κB-negative regulators in glioblastoma. (**a**–**c**) Infection of LN229 (**a**), BTSC23 (**b**) and BTSC233 (**c**) cells with pCHMWS lentivirus carrying FLAG-tagged KLF6-wt or KLF6-sv1 cDNA, or empty vector (EV), captured by immunoblotting. (**d–f**) qRT-PCR analysis of relative transcript expression of *TNIP1*, *TNIP2*, *NFKBIA* and *TNFAIP3* in LN229 (**d**), BTSC23 (**e**), BTSC233 (**f**) cells expressing EV, KLF6-wt or KLF6-sv1. (**g**) Immunoblotting of KLF6, TNFAIP3, NFKBIA in LN229 cells expressing EV, KLF6-wt or KLF6-sv1. (**h**) Schematic representation and position of KLF6 binding sites in the promoters of *TNIP1*, *TNIP2*, *NFKBIA* and *TNFAIP3* genes. Stars indicate binding sites validated by chromatin immunoprecipitation. (**i**) qRT-PCR analysis showing enriched binding of KLF6 to specific promoters in LN229 cells expressing EV, KLF6-wt or KLF6-sv1. The *SERPINA1* and *OLR1* gene promoters were positive and negative controls, respectively. Relative gene expressions are normalized to EV, representative qRT-PCR of three independent experiments. (**d–f**). Error bars represent mean±s.d.

To confirm that KLF6 is a transcriptional activator of the four NF-κB-negative regulators, LN229 glioblastoma cells and two brain tumor-derived stem-like cells (BTSCs) (BTSC23, BTSC233) were transduced with a lentivirus expressing empty vector (EV), KLF6-wt or the non-functional *KLF6* variant *sv1* as a negative control^24^ (Figures 3a–c and Supplementary Figures 3A–B). We confirmed the lack of nuclear localization by KLF6-sv1, as previously reported^24^ (Supplementary Figure 3C). RT–PCR confirmed that KLF6-wt, but not KLF6-sv1, induced expression of *NFKBIA* and *TNFAIP3*, and to a less extent, of *TNIP1* and *TNIP2*, in all the cell lines tested (Figures 3d–f). Correspondingly, increased NFKBIA and TNFAIP3 protein expression was detected by immunoblotting (Figure 3g), suggesting that NFKBIA and TNFAIP3 are the strongest NF-κB-negative regulators induced by KLF6. Chromatin immunoprecipitation in LN229 cells showed binding of KLF6-wt to all tested promoters, indicating that KLF6-wt can directly regulate the expression of the four NF-κB-negative regulators (Figures 3h and i).

**Figure 4:**
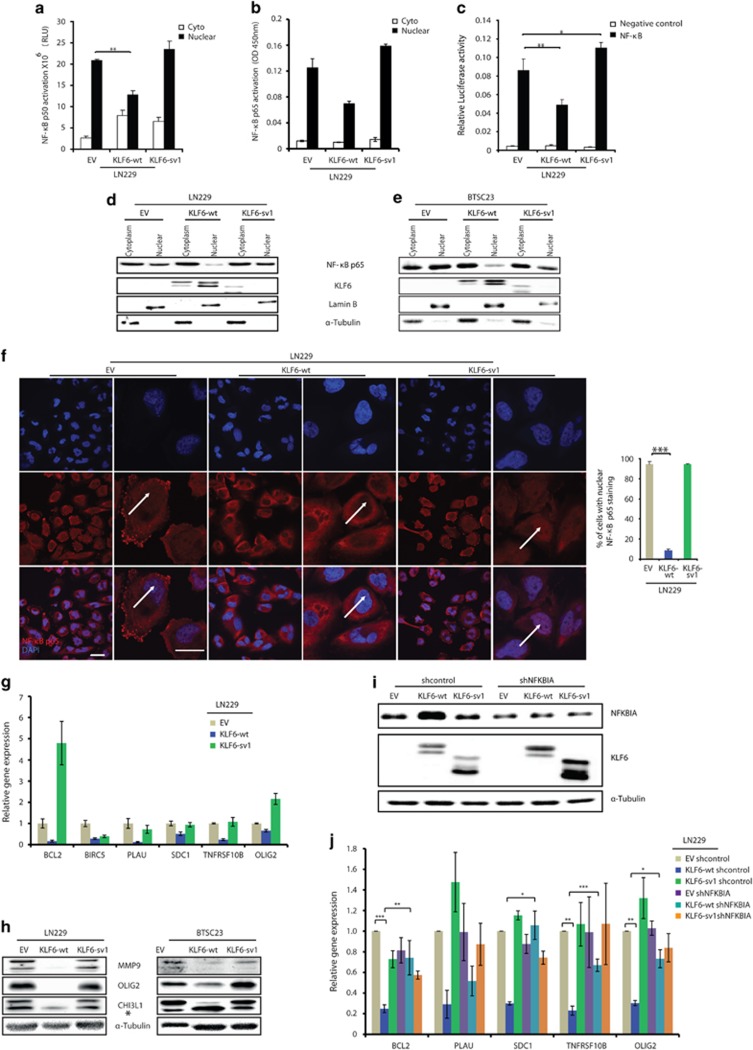
KLF6 inhibits NF-κB signaling in glioblastoma. (**a**–**b**) Analysis of NF-κB subunit p50 (**a**) and p65 (**b**) activation in nuclear and cytoplasmic extracts of LN229 cells expressing empty vector EV, KLF6-wt or KLF6-sv1. Data are normalized to protein concentration and are expressed as mean±s.d. (**c**) Luciferase assay of NF-κB subunit p65 in TNFα-stimulated LN229 cells expressing EV, KLF6-wt or KLF6-sv1. (**d**–**e**) Immunoblotting analysis for NF-κB p65 and KLF6 in nuclear and cytoplasmic extracts of TNFα-stimulated LN229 (**d**) and BTSC23 (**e**) cells expressing EV, KLF6-wt or KLF6-sv1. (**f**) Immunofluorescence staining of NF-κB subunit p65 in TNFα-stimulated LN229 cells expressing EV, KLF6-wt or KLF6-sv1. Nuclei were stained with 4',6-diamidino-2-phenylindole (DAPI). The right panel shows the corresponding quantification of nuclear:cytoplasmic ratio. The scale bar represents 30 μm (low mag) and 10 μm (high mag). (**g**) qRT-PCR analysis of relative transcript expression of NF-κB targets in LN229 cells. Relative gene expressions are normalized to EV, representative qRT-PCR of three independent experiments is shown. Error bars represent mean±s.d. (*n*=3 qPCR replicates) (**j**). (**h**) Immunoblotting of MMP9, OLIG2, YKL40 in LN229 and BTSC23 cells expressing EV, KLF6-wt or KLF6-sv1. *Represents an aspecific band. (**i**) Immunoblotting of NFKBIA and KLF6 in LN229 cells transduced as indicated. (**j**) qRT-PCR analysis of relative transcript expression of NF-κB targets in LN229 cells transduced as indicated. Error bars represent mean±s.d. of three independent experiments. *P<0.05, **P<0.01, ***P<0.005.

Next, we measured the effect of KLF6 on NF-κB pathway by looking at the activity of the canonical NF-κB subunits p50 and p65. In LN229 and BTSC233, KLF6-wt-mediated transcriptional activation of the NF-κB control genes was associated with reduced nuclear binding of the NF-κB subunits to a κB-responsive sequence (Figures 4a and b and Supplementary Figure 4A). Moreover, KLF6-wt, but not KLF6-sv1, reduced the expression of a NF-κB reporter construct (Figure 4c). Nuclear localization of p65 in LN229 and BTSC23 cells was reduced as a consequence of KLF6-wt expression as shown by immunoblotting of cytoplasmic and nuclear cell extracts (Figures 4d and e), and p65 immunostaining in LN229 cells (Figure 4f). Then, we measured the expression of a panel of NF-κB target genes previously implicated in glioblastoma by RT–PCR (Figure 4g and Supplementary Figures 4B and C).^32, 33, 34, 35, 36, 37, 38, 39^ In all tested cells, the majority of the genes were downregulated upon KLF6-wt overexpression, whereas KLF6-sv1 had no effect. Downregulation of NF-κB targets MMP9, OLIG2 and YKL40 was confirmed by western blot (Figure 4h). Finally, to demonstrate that KLF6-induced downregulation of NF-κB targets was due to upregulation of NF-κB-negative regulators, we silenced *NFKBIA* in LN229 cells previously transduced with EV, KLF6-wt or KLF6-sv1 (Figures 4i and j and Supplementary Figure 4D). As shown in Figure 4j, *NFKBIA* knockdown completely or partially rescued KLF6-wt effect on the majority of the tested genes; furthermore, although not always statistically significant, it seemed to prevent KLF6-mediated upregulation of the other NF-κB-negative regulators (TNIP1, TNIP2 and TNFAIP3, Supplementary Figure 4E), suggesting that NF-κB could mediate a positive feedback loop by downregulating its negative regulators as previously described.^40, 41^ Altogether, these results further confirm a role of KLF6 in NF-κB activation through transactivation of NF-κB control genes.

### KLF6 induces the expression of neural-like genes and inhibits the malignant phenotype of glioblastoma *in vitro*

**Figure 5:**
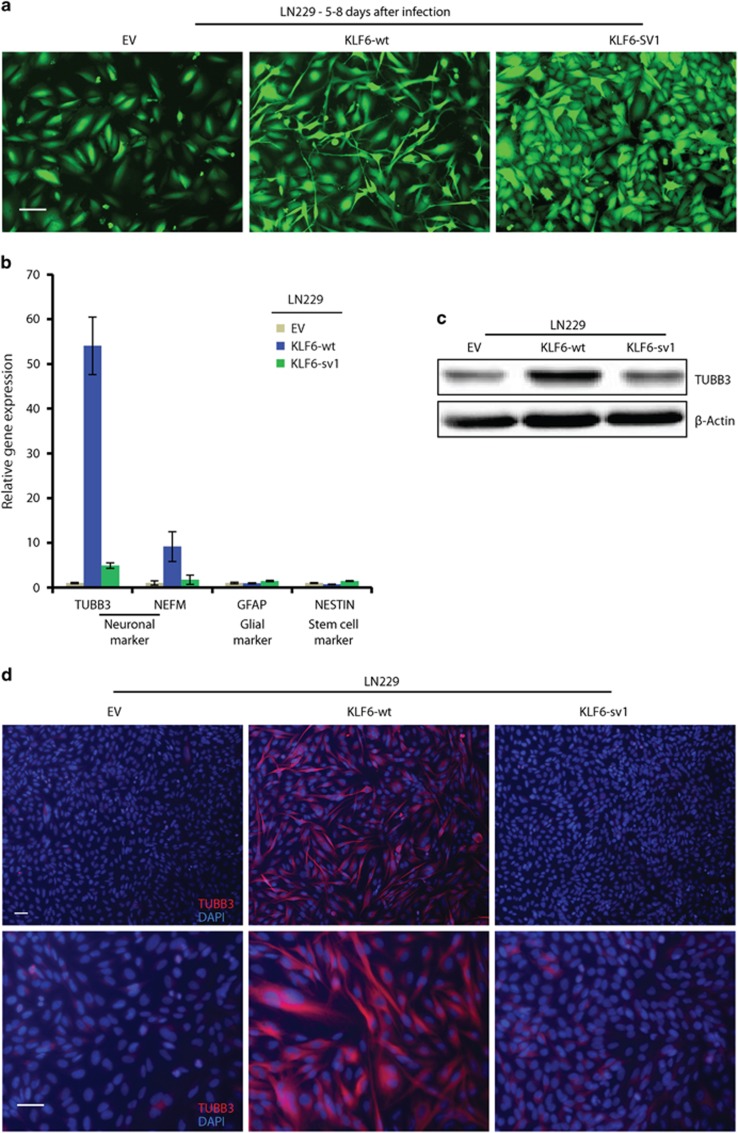
Wild-type KLF6 induces neural-like differentiation in LN229 cells. (**a**) Microphotographs of green fluorescent protein-positive LN229 cells expressing empty vector (EV), KLF6-wt or KLF6-sv1 after lentiviral infection. (**b**) qRT-PCR analysis of neuronal (*TUBB3* and *NEFM*), astrocytic (*GFAP*) and stem cell (*NESTIN*) markers in LN229 cells expressing EV, KLF6-wt or KLF6-sv1. (**c**) Immunoblotting of TUBB3 in LN229 cells expressing EV, KLF6-wt or KLF6-sv1. (**d**) Corresponding immunofluorescence staining for neuronal marker TUBB3 in LN229 cells expressing EV, KLF6-wt or KLF6-sv1. Nuclei were stained with DAPI. The scale bar represents 100 μm (**a**) or 50 μm (**d**). Relative gene expressions are normalized to EV, a representative qRT-PCR of three independent experiments is shown (**b**). Error bars represent mean±s.d.

LN229 cells expressing KLF6-wt had an elongated cell body reminiscent of neural cells (Figure 5a). These morphological changes were accompanied by upregulation of neuronal marker β3-tubulin (*TUBB3*) and neurofilament medium polypeptide (*NEFM*); by contrast, expression of glial marker glial fibrillary acidic protein (*GFAP*) and the stem cell marker *NESTIN* was not affected (Figures 5b–d). Immunoblotting and immunofluorescence confirmed high expression of TUBB3 upon KLF6-wt expression (Figures 5c and d). Moreover NESTIN was reduced while no changes in GFAP and two synaptic markers, PSD95 and SYP (Synaptophysin), were observed by immunostaining (Supplementary Figure 5A). These data suggests that KLF6-wt overexpression in LN229 activates the expression of some neuronal markers (TUBB3 and NEFM) although the differentiation appeared uncomplete due to lack of synaptic markers.

**Figure 6:**
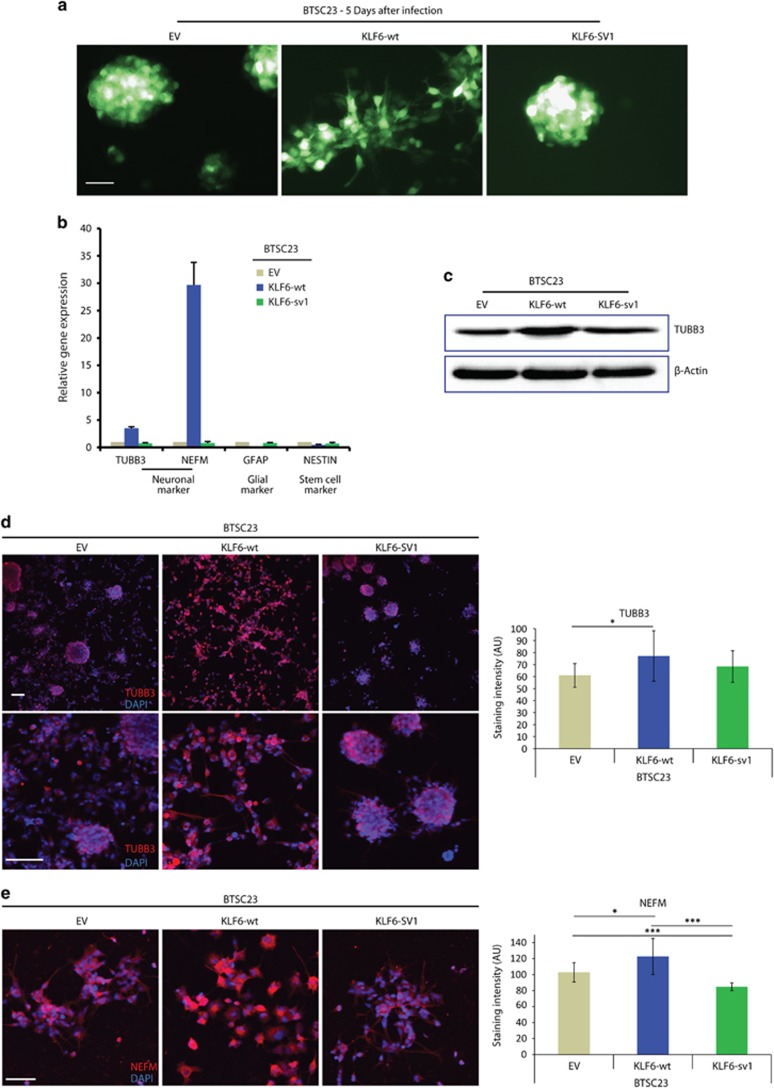
Wild-type KLF6 induces neural-like differentiation in BTSC23 cells. (**a**) Microphotographs of GFP-positive BTSC23 cells expressing empty vector (EV), KLF6-wt or KLF6-sv1, after lentiviral infection. (**b**) qRT-PCR analysis of neuronal (*TUBB3, NEFM*), astrocytic (*GFAP*) and stem cell (*NESTIN*) markers in BTSC23 cells expressing EV, KLF6-wt or KLF6-sv1. (**c**) Immunoblotting of TUBB3 in BTSC23 cells expressing EV, KLF6-wt or KLF6-sv1. (**d**) Immunofluorescence staining and relative quantification for TUBB3 in BTSC23 cells expressing EV, KLF6-wt or KLF6-sv1. Nuclei were stained with DAPI. (**e**) Immunofluorescence staining using Tyramide Signal Amplification and relative quantification for NEFM in BTSC23 cells expressing EV, KLF6-wt or KLF6-sv1. Nuclei were stained with DAPI. *P<0.05, ***P<0.001. The scale bar represents 50 μm (**a**, **e**) or 100 μm (**d**). Relative gene expressions are normalized to EV, a representative qRT-PCR of three independent experiments is shown (**b**). Error bars represent mean±s.d.; *n*=10 in **d**–**e**.

KLF6-wt overexpression in two BTSCs (BTSC23, BTSC233) led to morphological changes similar to LN229 (Figure 6a and Supplementary Figure 6A). RT–PCR analyses in BTSC23 cells showed increased *TUBB3* and *NEFM* expression, whereas *GFAP* expression was slightly reduced and *NESTIN* expression was unaffected (Figure 6b). Similar results for *TUBB3* and *GFAP* were obtained in BTSC233 cells (Supplementary Figure 6B), whereas *NEFM* was expressed at very low levels in all the samples (data not shown). TUBB3 and NEFM increase in BTSC23 was confirmed at the protein level (Figures 6c–e, Supplementary Figures 6C and D). Interestingly, in BTSC233, but not in BTSC23, cells higher levels of GFAP were detected by immunostaining (Supplementary Figure 5B and Supplementary Figure 6E), suggesting that glial markers could also be regulated in a cell-dependent context. PSD95 and SYP were also upregulated upon KLF6-wt expression in BTSC233 and BTSC23 cells, respectively (Supplementary Figure 5B and Supplementary Figures 6F and G). Overall, these data indicate that, although the expression of specific neural markers can differ in various glioblastoma cells, KLF6-wt and ensuing NF-κB inhibition appear to induce a similar pattern of expression of neural genes and loss of stem marker NESTIN, suggesting the activation of an aberrant differentiation program.

**Figure 7:**
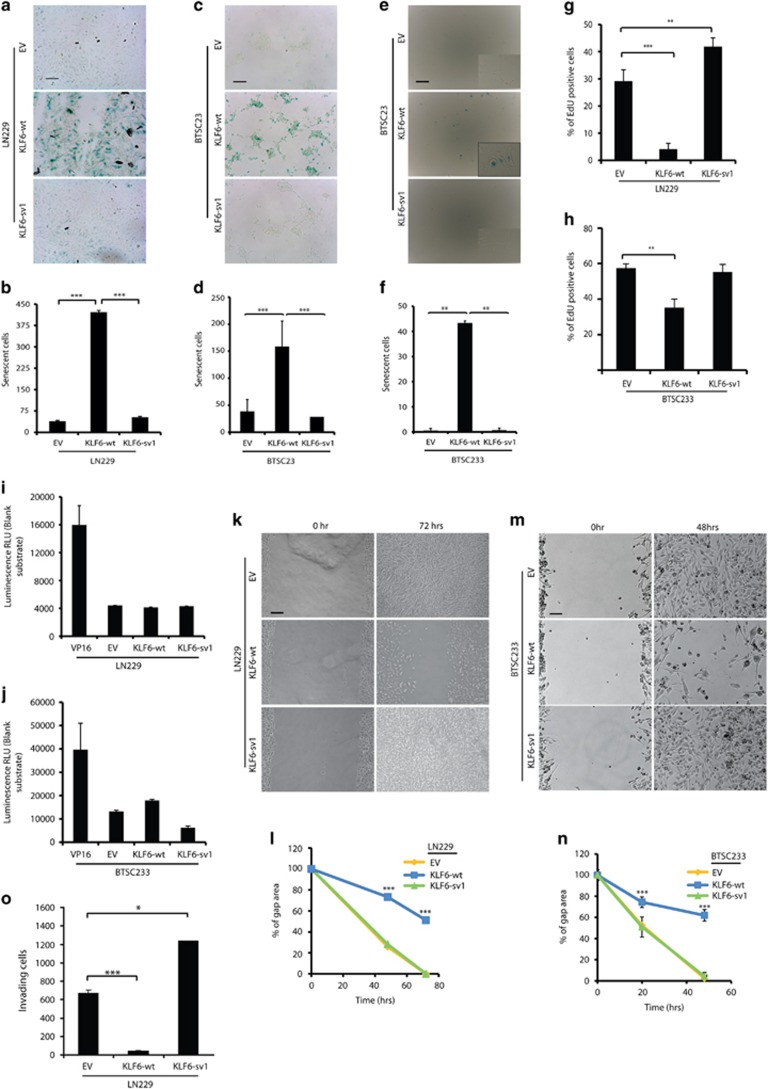
KLF6 inhibits the malignant phenotype of glioblastoma *in vitro.* (**a–f**) Cellular senescence in LN229 (**a**), BTSC23 (**c**), BTSC233 (**e**) cells expressing empty vector (EV), KLF6-wt or KLF6-sv1 assessed by senescence-associated β-galactosidase detection and corresponding quantification of stained cells in LN229 (**b**), BTSC23 (**d**), BTSC233 (**f**). (**g**–**h**) Quantification of EdU staining in LN229 (**g**) and BTSC233 (**h**) cells expressing EV, KLF6-wt or KLF6-sv1. (**i**–**j**) Analysis of cell death apoptotic activity in LN229 (**i**) and BTSC233 (**j**) cells. Readings were normalized to blank and are expressed as mean±s.d. (**k**–**n**) Scratch assay in LN229 (**k**) and BTSC233 (**m**) cells expressing EV, KLF6-wt or KLF6-sv1, and corresponding quantification in LN229 (**l**) and BTSC233 (**n**) cells. (**o**) Quantification of invading LN229 cells expressing EV, KLF6-wt or KLF6-sv1 in a matrigel invasion assay. *P<0.05, **P<0.01, ***P<0.001. Scale bars represent 100 μm (**a**, **c**, **e**), 200 μm (**k**) or 50 μm (**m**). Error bars represent mean±s.d. (*n*=3).

Prolonged expression of KLF6-wt, but not KLF6-sv1, induced a senescent-like phenotype in both LN229 and BTSC23 cells, highlighted by β-galactosidase staining (Figures 7a–f). Cell cycle analysis revealed accumulation of cells in phase G1–G0 and concomitant reduction of cells in phases S and G2/M, upon KLF6-wt expression (Supplementary Figure 7A). In contrast, KLF6-sv1 overexpression prolonged S-phase (Supplementary Figure 7A). In BTSC23 cells, KLF6-wt overexpression led to accumulation of cells in G2/M (Supplementary Figure 7B). Consistent with the G1 arrest observed in LN229 cells, KLF6-wt overexpression led to upregulation of *CDKN1A* expression in LN229 and BTSC23 cells (Supplementary Figures 7C–D). In accordance with a reduced number of cells in S-phase, KLF6-wt reduced cell growth in both LN229 and BTSC23 cells (Figures 7g and h). KLF6-wt cells appeared less viable compared with control or KLF6-sv1-expressing cells (Supplementary Figures 7E and F) suggesting that KLF6-wt could cause cell death. Only a small increase in apoptosis was observed in BTSC233 but not in LN229 cells upon KLF6-wt overexpression (Figures 7i and j). Interestingly, in BTSC233 cells, KLF6-sv1 reduced apoptosis supporting previous studies that proposed KLF6-sv1 as an oncogenic variant, inhibiting pro-apoptotic factors.^25, 42, 43^ Finally, KLF6-wt but not KLF6-sv1 overexpression reduced cell migration and invasion in LN229 and BTSC233 cells (Figures 7k–o and Supplementary Figure 7G). Interestingly, LN229 cells transduced with KLF6-sv1 were more proliferative and invasive compared with control cells (Figures 7a and o), supporting KLF6-sv1 oncogenic role.

### Wild-type KLF6 delays gliomagenesis *in vivo*

**Figure 8:**
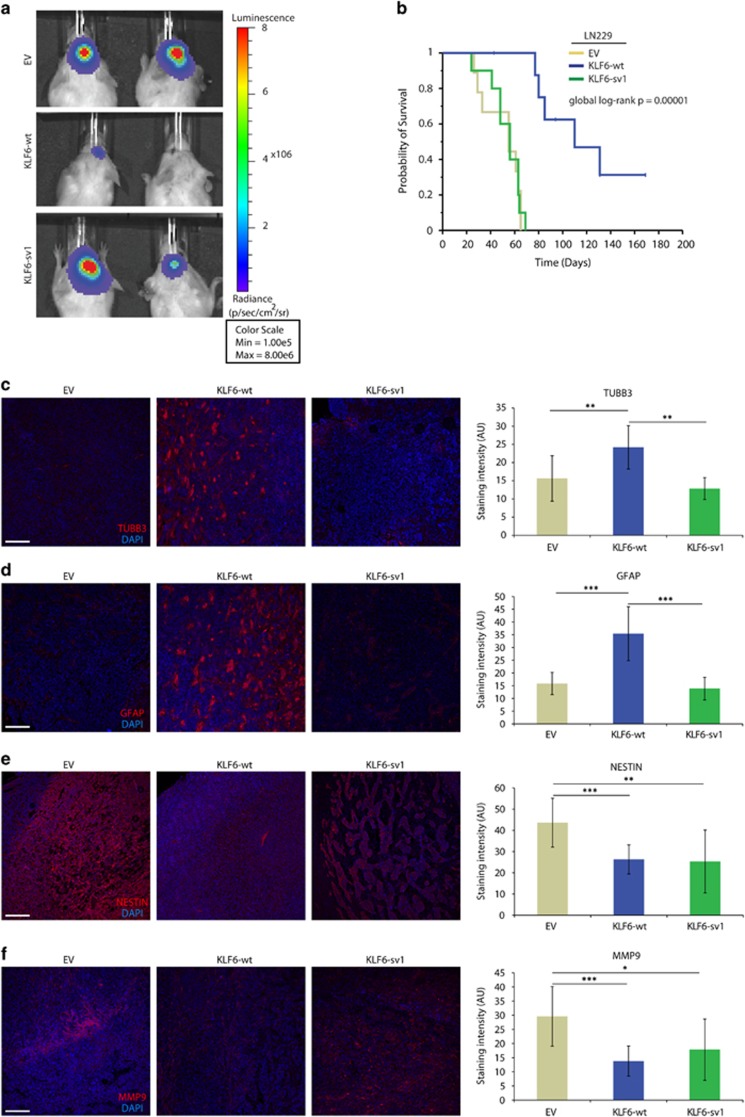
KLF6 delays gliomagenesis *in vivo*. (**a**) Representative *in vivo* BLI analysis in NOD/SCID mice 3 weeks after intracranial injection of LN229 cells infected with lentiviral vector carrying KLF6-wt cDNA, KLF6-sv1 cDNA or EV, and pCHMWS-fLuc to express a luciferase gene for BLI detection. (**b**) Kaplan–Meier estimates of survival in animal subgroups injected as described in **a** (10 animal per group). (**c–f**) Immunofluorescence staining and relative quantification for TUBB3 (**c**), GFAP (**d**), NESTIN (**e**), MMP9 (**f**), in tumors resulting from intracranial injection of LN229 cells injected as described in **a**. Nuclei were stained with DAPI. *P<0.05, **P<0.01, ***P<0.001. Scale bars represent 100 μm (**c**–**f**). Error bars represent mean±s.d.; *n*=9 in **b**, *n*=10 in **c**–**f**.

Next, we examined whether KLF6-wt and KLF6-sv1 affected tumor formation *in vivo*. Ectopic expression of KLF6-wt attenuated tumor growth as evidenced by reduced bioluminescence imaging (BLI analysis) 3 weeks after cell injection (*P*=0.001, *t*-test) (Figure 8a). Moreover, KLF6-wt overexpression improved survival (Figure 8b), although tumors eventually developed in all the three groups and appeared similar in size (Supplementary Figure 8A). Analysis of the proliferation marker Ki67 (MIB1) did not reveal difference in proliferation, suggesting that perhaps, at the experimental end point the more proliferative cells have been selected and enriched (Supplementary Figure 8B). Supporting our hypothesis that KLF6 regulates NFKBIA expression, NFKBIA was moderately upregulated in KLF6-wt tumors (Supplementary Figure 8C). As observed *in vitro*, KLF6-wt tumors expressed lower levels of NESTIN and appeared more differentiated as indicated by higher levels of TUBB3 and GFAP (Figures 8c–e). In addition, MMP9 was reduced consistently with our previous observation (Figure 8f). Our *in vivo* results point toward KLF6 as a tumor suppressor in glioblastoma inhibiting NF-κB activity, causing loss of stemness and promoting differentiation.

## Discussion

In this study we have identified a previously unappreciated NF-κB regulatory pathway that involves transactivation of NF-κB-negative regulatory genes by the tumor suppressor KLF6 in glioblastoma. These findings reinforce prior evidence of a role of the NF-κB pathway in glioma progression.^38, 44, 45^ Distinct from others, our proposed mechanism of NF-κB deregulation does not involve mutations of NF-κB pathway components.^2^

Our data support prior studies indicating that KLF6 is a tumor suppressor in human cancer^11, 42^ and provide a mechanism for its function in glioblastoma. Moreover, in agreement with a previous study in hepatocellular carcinoma,^13^ we found that loss of one *KLF6* allele significantly shortens both progression-free and overall patient survival. We also observed that the remaining *KLF6* allele is rarely mutated in glioblastoma, as previously reported.^22^ Altogether, these data suggest that *KLF6* exhibits haploinsufficiency in glioblastoma. However, as all the samples analyzed lacked KLF6 expression, it is possible that other mechanisms are involved in suppressing KLF6 expression. In accordance with this hypothesis, KLF6 has been recently shown to be silenced by increased trimethylation of histone H3 at Lys9 (H3K9me3) levels in dedifferentiated liposarcomas.^46^ As such, further studies would be required to determine whether additional epigenetic mechanisms silence KLF6 in glioblastoma and/or whether they are alternative to genomic alterations.

Co-activator roles of KLF6 in inducing NF-κB targets were recently proposed.^47, 48^ Although these data diverge from our proposed model, it is possible that KLF6 might function as NF-κB activator or inhibitor in different tissues and/or in response to different stimuli. As the majority of our experiments were performed in absence of stimulation, it is possible that NF-κB activation by other factors might counteract KLF6-mediated effect. More studies would be necessary to further investigate the relationship between KLF6 and NF-κB in different cell models and condition.

Consistent with previous data,^15^ we show that KLF6 induces *CDKN1A* expression in LN229 cells and concomitantly accumulates cells in the G1 phase. In BTSC23 cells, KLF6-wt caused accumulation of cells in G2 phase, which could reflect the different nature of this tumor cell type. This is consistent with previous data in colon cancer cell lines showing that the number of cells arresting in G1 or G2 can be cell type dependent due to regulation by different checkpoint mechanisms.^49^ As CDKN1A inhibits both G1 and G2 cell cycle phase progression by binding to and inhibiting CDK2 and CDK1,^50^ our observation that KLF6-wt mediates G2 arrest in BTSC23 cells is also consistent with the induction of *CDKN1A* by KLF6. Hoeferlin *et al.*^49^ suggest that the lower level of *CDKN1A* expression in HCT116 could be insufficient to cause G1 arrest. Interestingly, we also observed a lower induction of *CDKN1A* in BTSC23, which accumulated in G2 upon KLF6-wt overexpression. However, it is also possible that CDKN1A-independent mechanisms leading to accumulation of cells in G2 might be activated in response to KLF6-wt.

We observed decreased cell viability in LN229-KLF6-wt cells, suggesting that KLF6 might induce cell death; however, only a small increase in apoptosis was observed upon KLF6 overexpression in BTSC233 cells, which suggests the involvement of additional cell death mechanisms such as mitotic catastrophe, which has been associated with cell senescence.^51^

We show that KLF6 overexpression often reduced NESTIN and increased neural genes expression in glioblastoma cells and LN229-KLF6-wt derived tumors, suggesting that KLF6-wt could reduce stemness and induce neural differentiation. The enhanced ability of KLF6-wt expressing cells to activate an aberrant/incomplete neural-like differentiation program is in line with a previously reported role of NF-κB in mesenchymal differentiation of glioblastoma-initiating cells.^52^ Interestingly, blockade of NF-κB in these cells is also associated with increased senescence, similarly to what we observed in our study.^52^ One could argue that senescence occurs as a consequence of activating an aberrant KLF6-mediated neural differentiation pathway in glioblastoma cells growing under proliferative conditions. Higher levels of GFAP protein, but not RNA, were observed upon KLF6 overexpression in BTSC233 cells. As this increase was not accompanied by upregulation of the *GFAP* transcript (Supplementary Figure 5B), it is possible that KLF6 overexpression might cause GFAP posttranslational modifications affecting its stability. Interestingly, GFAP phosphorylation has been shown to regulate its stability.^53^ More studies would be necessary to investigate the role of KLF6 in GFAP protein regulation. We show that KLF6 regulated NF-κB via NF-κB regulators requires KLF6 localization into the nucleus and binding to their promoters. In agreement, the splice variant sv1 lacking the nuclear localization domain does not affect NF-κB activation. Interestingly, overexpression of KLF6-sv1 leads to a significant increase in the percent of cells in S-phase, cellular proliferation and cell invasion, consistent with the proposed role of KLF6-sv1 as an oncogenic variant, which drives metastasis and is associated with poor survival in prostate cancer.^25^ Our data suggest that KLF6-sv1-mediated oncogenic functions do not require translocation into the nucleus and repression of NF-κB-negative regulators. Further studies would be required to characterize the mechanism of KLF6-sv1 function in tumorigenesis and metastasis.

Collectively, our data are the first to identify KLF6 as a negative regulator of NF-κB and indicate that disruption of this system can occur by loss of function of *KLF6* through haploinsufficiency. Our findings could potentially translate the use of NF-κB inhibitors in combination to drugs that allow KLF6 re-expression. Interestingly, it was shown that targeting the KLF6 regulator FOXO1 and EGFR with trifluoperazine hydrochloride, a FOXO1 nuclear export inhibitor, and erlotinib, a small-molecule inhibitor of EGFR signaling, leads to KLF6 re-expression.^25^ In the future, it would be interesting to test the effect of these drugs, alone or in combination with NF-κB inhibitors, on glioblastoma cell proliferation and invasion, *in vitro* and *in vivo.*

## Materials and methods

### Tumor samples and patients

Samples were collected at the University of Freiburg and the University of Alabama at Birmingham (UAB), under institutional review board (IRB)-approved guidelines. Written informed consent was obtained from all patients. About 537 glioblastoma samples from TCGA (http://cancergenome.nih.gov/) were used as discovery set. Differences in sample sizes for the different analyses reflect the availability of patient data for the different inferred molecular levels (messenger RNA, copy number and so on), platforms and incomplete overlap between these data sets. Raw Affymetrix Genome-Wide Human SNP Array 6.0 and Agilent Human Genome CGH Microarray 244A gene dosage data, Affymetrix Human Genome U133 Plus 2.0 Array and Agilent 244K Custom Gene Expression data, and Affymetrix Human Exon 1.0 ST Array exon-specific expression data, and clinical data were retrieved from the TCGA upon National Human Genome Research Institute (NHGRI) approval and pre-processed for downstream analyses.

### Cell lines and cell culture

LN229 glioblastoma cells were obtained from the ATCC and grown according to basic cell culture techniques. Patient-derived glioblastoma stem cells (BTSCs) were prepared from tumor specimens under IRB-approved guidelines and grown as previously described.^54^ All cells were mycoplasma-free.

### Gene copy number variation analysis

Gene-level copy number variation was estimated using the CBS algorithm^55^ from the 'snapCGH' package for R. Gene dosage segments were classified as chromosomal ‘gain’ or ‘loss’ if the absolute value of the predicted dosage was more than 0.75 times the interquartile range of the difference between observed and predicted values for each region. Copy number variation data processed using the GISTIC2 algorithm^56^ were retrieved from the Broad Institute at http://gdac.broadinstitute.org/runs/analyses__2012_03_21/data/GBM/20120321/. Genes mapped onto the human genome coordinates using the University of Santa Cruz Biotechnology (Dallas, TX, USA) (UCSC) cgData HUGO probeMap were visualized using the UCSC Cancer Genomics Browser (https://genome-cancer.ucsc.edu/).

### Quantitative real-time PCR

RNA was prepared as described before.^54^ Quantitative real-time PCR (qRT-PCR) was performed in triplicate using SYBR Green or pre-validated TaqMan assays (Applied Biosystems, Thermo Fisher, Waltham, MA, USA): KLF6-wt: HKLF6E2E3wt, KLF6-sv1: HS01062724, CHI3L1: HS00609691, CDKN1A: HS00355782. Primer sequences are listed in Supplementary Table 1. Experiments were validated three times, a representative experiment (mean refers to technical replicates) or the average of three experiments was shown.

### Immunoblotting and immunofluorescence

Antibodies for immunoblotting and immunostaining are listed in Supplementary Table 5. Secondary labeling of NEFM antibody was performed with Tyramide Signal Amplification Kit (PerkinElmer, Waltham, MA, USA) according to the manufacturer’s instructions. Samples were counterstained with DAPI. Pictures were acquired using an Axiovert Microscope (Zeiss, Oberkochen, Germany) and FSL confocal microscope (Olympus, Tokyo, Japan). Axiovision AXIOVS40 V4.8.0.0 (Carl Zeiss, Oberkochen, Germany) or Fluoview FV10-ASW3.1 (Olympus) software was used for image processing and quantifications.

### Vectors and lentiviral infection

Lentiviral infections were performed as previously described^54^ using the lentiviral vector pCHMWS (kind gift from Veerle Baekelandt, University of Leuven) expressing KLF6-wt, KLF-sv1 or EV. Knockdown of NFKBIA was obtained with a shRNA lentiviral vector (pGIPZ, Open Biosystem Clone ID _V3LHS_410687). Cloning primers are listed in Supplementary Table 3.

### Chromatin immunoprecipitation

Promoter analysis was performed with the MatInspector software (www.Genomatix.de). Primers were designed using the Primer3 software (http://bioinfo.ut.ee/primer3-0.4.0/primer3/) and are listed in Supplementary Table 2. Chromatin immunoprecipitation was performed three times as previously described^57^ using KLF6 antibody (R-173, Santa Cruz Biotechnology) or normal rabbit immunoglobulins (Santa Cruz Biotechnology). Eluted DNA was analyzed by absolute qRT-PCR. Amplified product was expressed as a percentage of the input for each condition. The *SERPINA1* and *OLR1* gene promoters were positive and negative controls, respectively.^58^

### Mutational analysis

*KLF6* sequence analysis was carried out on a 3730 DNA Analyzer (Applied Biosystems). Primer sequences are listed in Supplementary Table 4.

### Nuclear and cytoplasmic extracts, and NF-κB DNA binding assay

Cytoplasmic and nuclear extracts were prepared using a Nuclear Extract Kit (Active Motif, La Hulpe, Belgium) and processed using the TransAM assay p50 and p65 kits (Activ Motif, La Hulpe, Belgium) according to the manufacturer's instructions.

### Luciferase assay

Luciferase analysis was performed using the Luciferase Reporter Assay System (Promega, Madison, WI, USA) according to the manufacturer's instructions. About 2.5 × 10^5^ cells were seeded in six-well plates prior to transfection with NF-κB-responsive vector (*3x*k*B.luc*) or control vector together with renilla luciferase vector. Six independent transfections were done using Lipofectamine according to the manufacturer’s instruction (Invitrogen, Thermo Fisher). Four to six hours after transfection, cells were infected for 72 h and then stimulated with 10 ng/ml recombinant human TNFα (R&D systems, Minneapolis, MN, USA) for 6 h. Luciferase activity was determined using a Thermo Scientific Appliskan luminometer (Termo Fisher). All data were reported relative to luciferase activity (*firefly*/*renilla*).

### Cell viability assay

Cells were harvested 5 days after lentiviral transduction and 2000 cells per well were seeded in 96-well plates. Proliferation was measured at 24-h intervals up to 6 days (LN229) or at 0, 1, 4, 5, 7, 8 days (BTSC23) by (3-(4,5-dimethylthiazol-2-yl)-2,5-diphenyltetrazolium bromide (MTT)) conversion (Roche, Mannheim, Germany) at 550/700 nm on a plate reader, according to the manufacturer’s instructions. Assays were conducted in six replicates in two independent experiments.

### EdU cell proliferation assay

Cell proliferation was assessed using the EdU-Click594 Cell Proliferation Imaging Kit (Baseclick GmbH, Neuried, Germany) according to the manufacturer's instructions. About 2 × 10^4^ cells were seeded on glass coverslips (LN229) or laminin-coated glass coverslips (BTSCs) in a 24-well plate. Pictures were acquired using an Axiovert Microscope (Zeiss). Assays were conducted in triplicates in two independent experiments.

### Cell cycle analysis

For flow cytometry analysis cells were infected as described above. At 24-h intervals, floating and attached cells were harvested and fixed with 70% ethanol for 1 h at 4 °C. Fixed cells were treated with 0.2 mg/ml RNase A (Sigma, St Louis, MO, USA) for 1 h at 37 °C, stained with 10 μg/ml propidium iodide (PI; Sigma) and analyzed on a FACScan flow cytometer (Becton Dickinson, Franklin Lakes, NJ, USA). Percentages of cells in G0–G1, S and G2–M phases were determined (Flow Jo, Ashland, OR, USA). Three independent experiments were performed.

### Senescence assay

Senescence was assessed using the Senescence Detection kit (Calbiochem, Darmstadt, Germany) according to the manufacturer’s instructions. Cells were seeded in triplicates, fixed and stained 5 days upon lentiviral transduction. Pictures were acquired using an Axioimager 2 Microscope (Zeiss). β-galactosidase-positive cells were counted and expressed as a percentage of total cells. The assay was validated in two independent experiments.

### Caspase 3/7 activity assay

For caspase assay, 2000 cells per well were seeded in 96-well plates (Promega). Transduced and positive control cells (treated with 2.5 μm VP16 (Etoposide) for 24 h) were seeded in triplicates and incubated with Caspase 3/7 ^R^ Glo reagent and analyzed in a plate-reading luminometer as per manufacturer’s instruction. The assay was validated in two independent experiments.

### Migration and invasion assays

Migration and invasion assays were performed as described before.^54^ Images of migrating cells were taken every 24 h. For BTSC233 cells, laminin-coated (Invitrogen, Thermo Fisher, 4 μg/ml) 60 mm dishes containing a culture insert (Ibidi, Martinsried, Germany) were used. For invasion assay, 2.5 × 10^4^ cells were seeded in four replicates in the upper compartment. Pictures were acquired using an Axioimager 2 Microscope (Zeiss). The assays were validated in two independent experiments.

### Intracranial injection and bioluminescence analysis

Intracranial injections were performed in NOD/SCID mice (Charles River Laboratory) in accordance with the directive 86/609/EEC of the European Parliament, following approval by regional authorities, as described before.^59^ Mice were randomized in experimental groups by a blinded operator. Sample size in each group (10) was chosen based on one-way analysis of variance to give 80% power to detect significant difference with three groups. Animals were monitored daily until the development of neurological symptoms by a blinded operator. Animals which did not show tumors were excluded from the analysis. Bioluminescence imaging was conducted with a CCD camera (IVIS, Xenogen, Caliper life Science, Maiz, Germany). The data were expressed as photon-flux (photons/sec/cm^2^/steradian).

### Histological analysis and immunohistochemistry

Histology was performed as previously described.^60, 54^ Sections were incubated with primary antibodies listed in Supplementary Table 5. Nuclei were counterstained with DAPI. Pictures were acquired using a FSL confocal microscope (Olympus) and AxioImager 2 (Zeiss). Fluoview FV10-ASW3.1 (Olympus) and Adobe Photoshop CS5 software (San Jose, CA, USA) were used for image processing and quantifications. Quantification of Ki67 staining was done using IHC profiler in ImageJ.^61^

### Statistical analyses

Linear regression analyses and graphical model validation were executed using R software (Vienna, Austria). Scatterplots and locally weighted least squares smooths were used to confirm the suitability of linear regression analyses, and statistical significance was assessed according to the *P*-value for the estimated slope of the regression line. Survival curves were estimated by the Kaplan–Meier product-limit method, and qsurvival distributions were compared across the groups using the log-rank test. Univariate and multivariate Cox proportional hazards regression analyses were carried out with progression-free survival or overall survival as the dependent variable and *KLF6* gene dosage or KLF6 gene expression as the primary predictor. Two-way contingency table analysis, unpaired *t*-test and Wilcoxon rank-sum test were used as appropriate. Odds ratios in the two-way contingency table analysis and 95% confidence intervals were computed using Woolf’s method for variance estimation. Comparison of treatment was done by paired two-ways Student’s *t*-test. *P*-values <0.05 were considered significant. Error bars represent s.d.
