# Supplementary figures and images for: KLF6 depletion promotes NF-κB signaling in glioblastoma
Source: Oncogene. 2017 Feb 6;36(25):3562–75. doi: 10.1038/onc.2016.507 (PMC5485221; doi:10.1038/onc.2016.507)

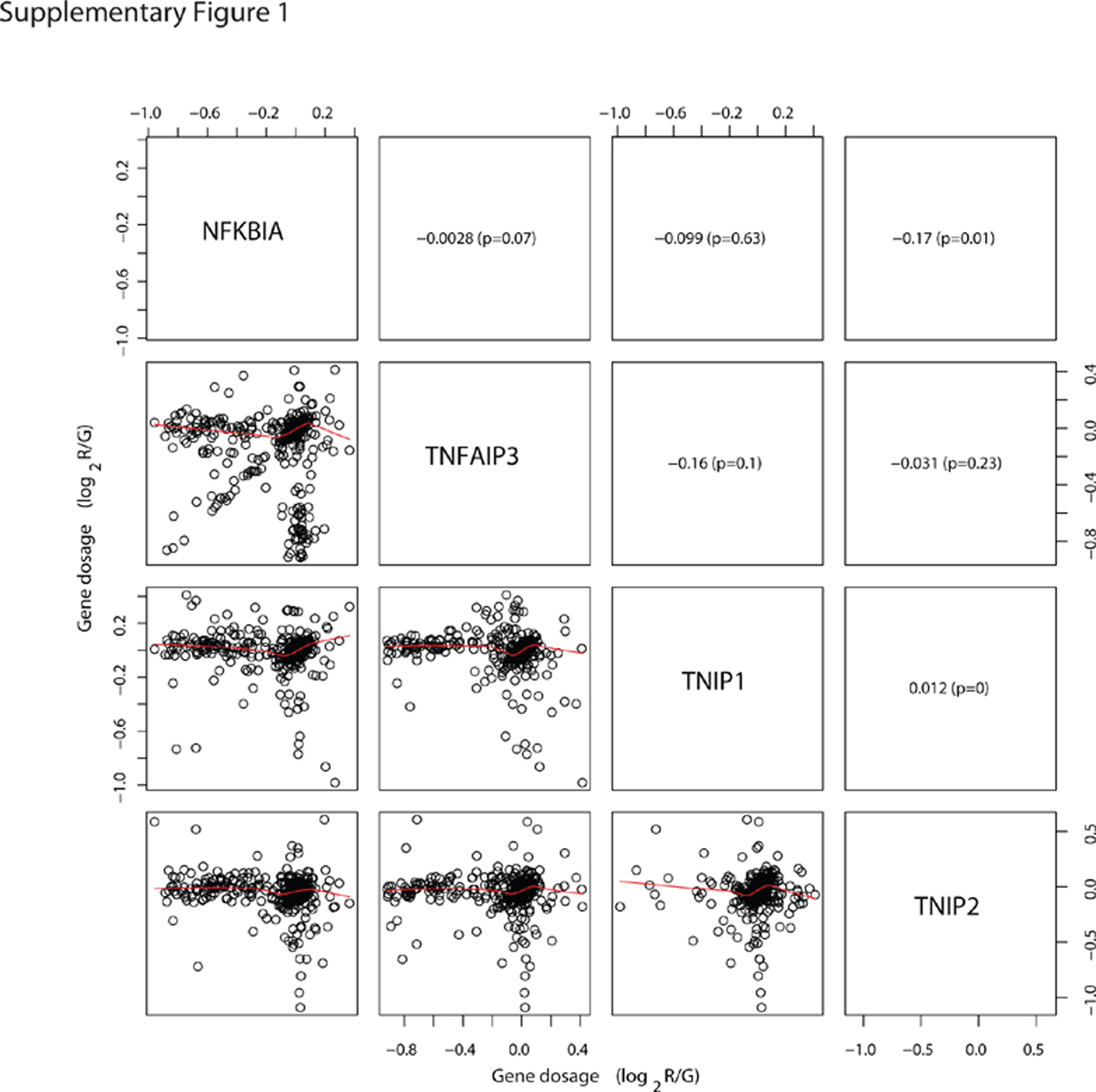

Supplement: Supplementary Figure 1 [file onc2016507x1.tif]

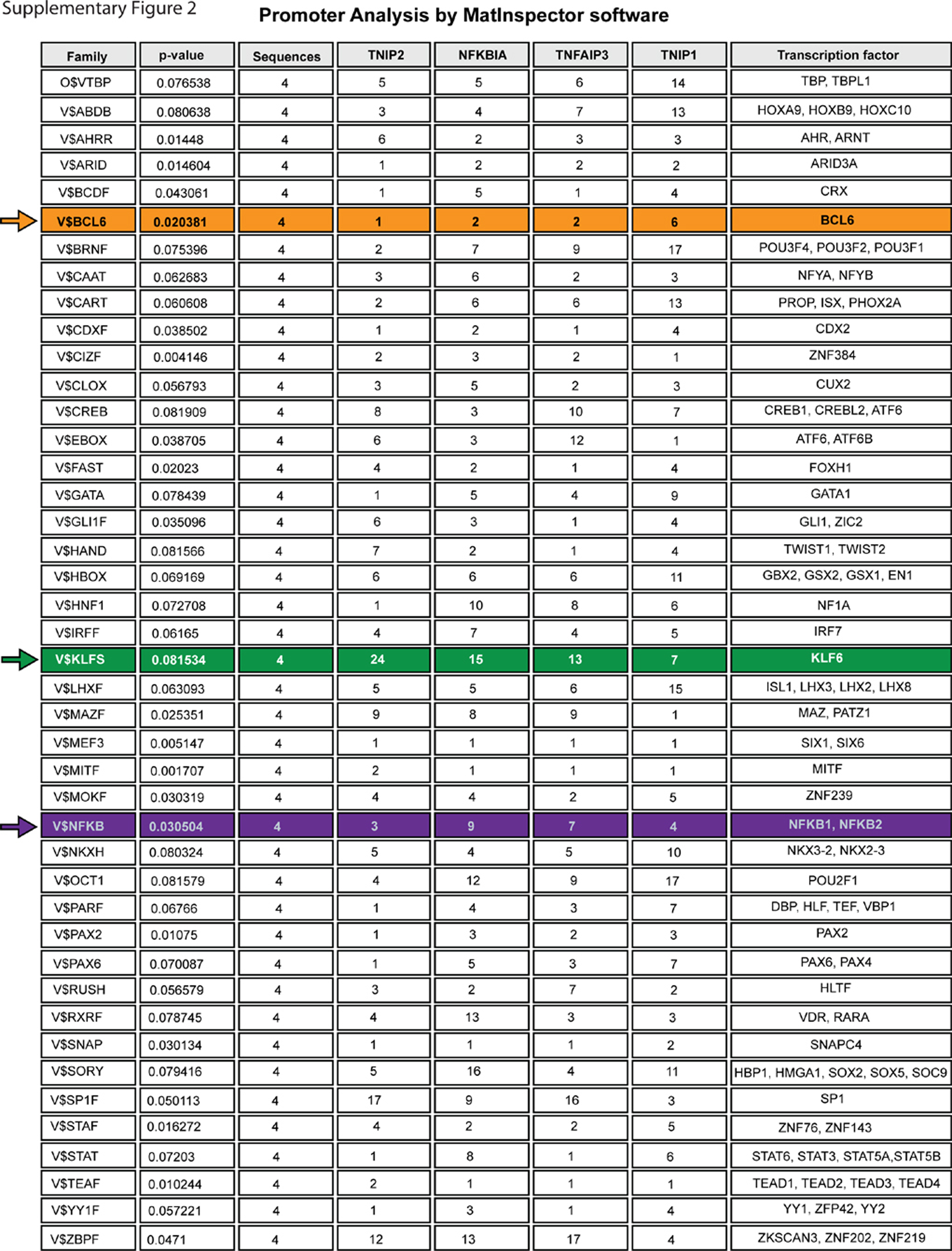

Supplement: Supplementary Figure 2 [file onc2016507x2.tif]

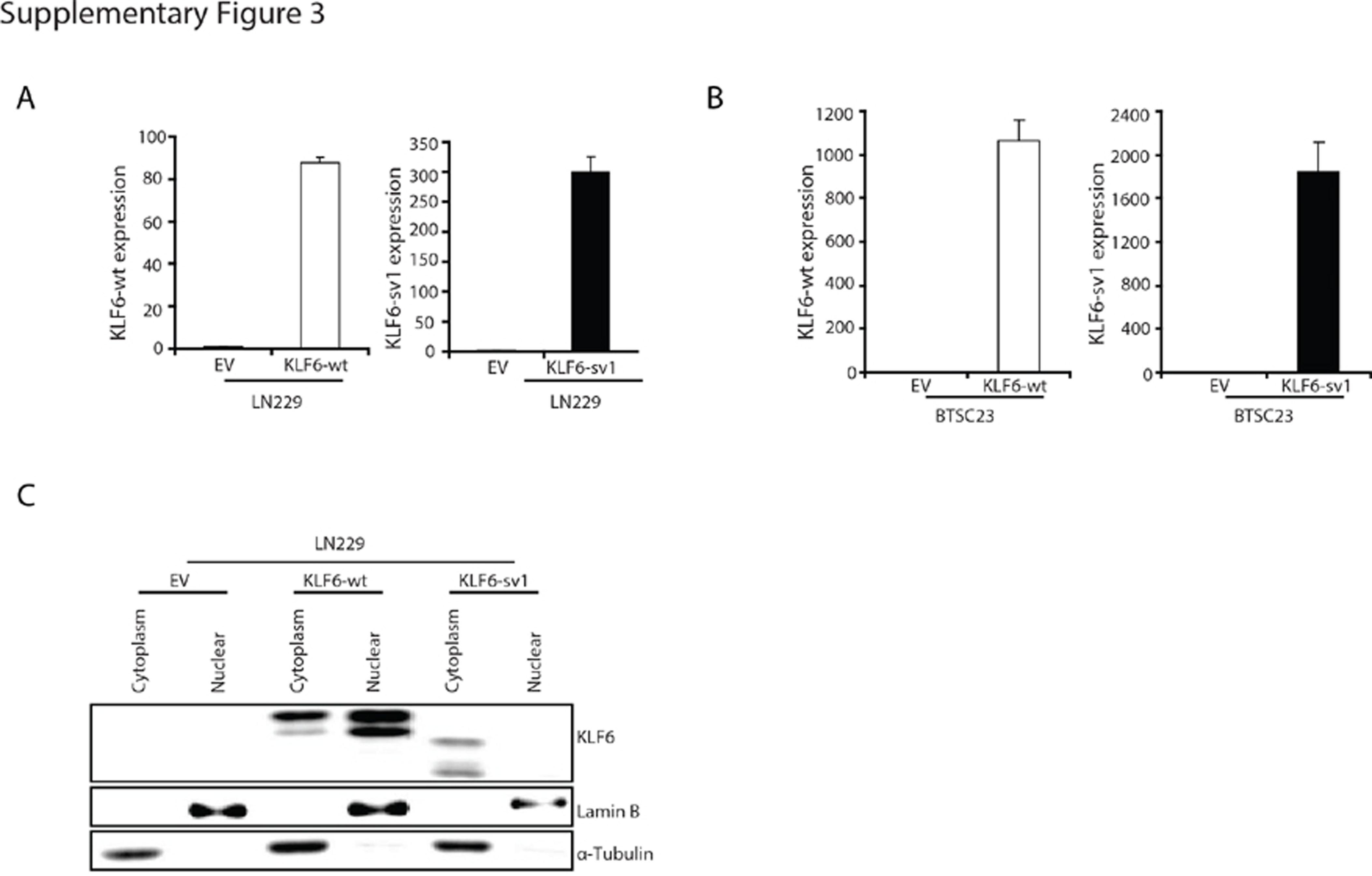

Supplement: Supplementary Figure 3 [file onc2016507x3.tif]

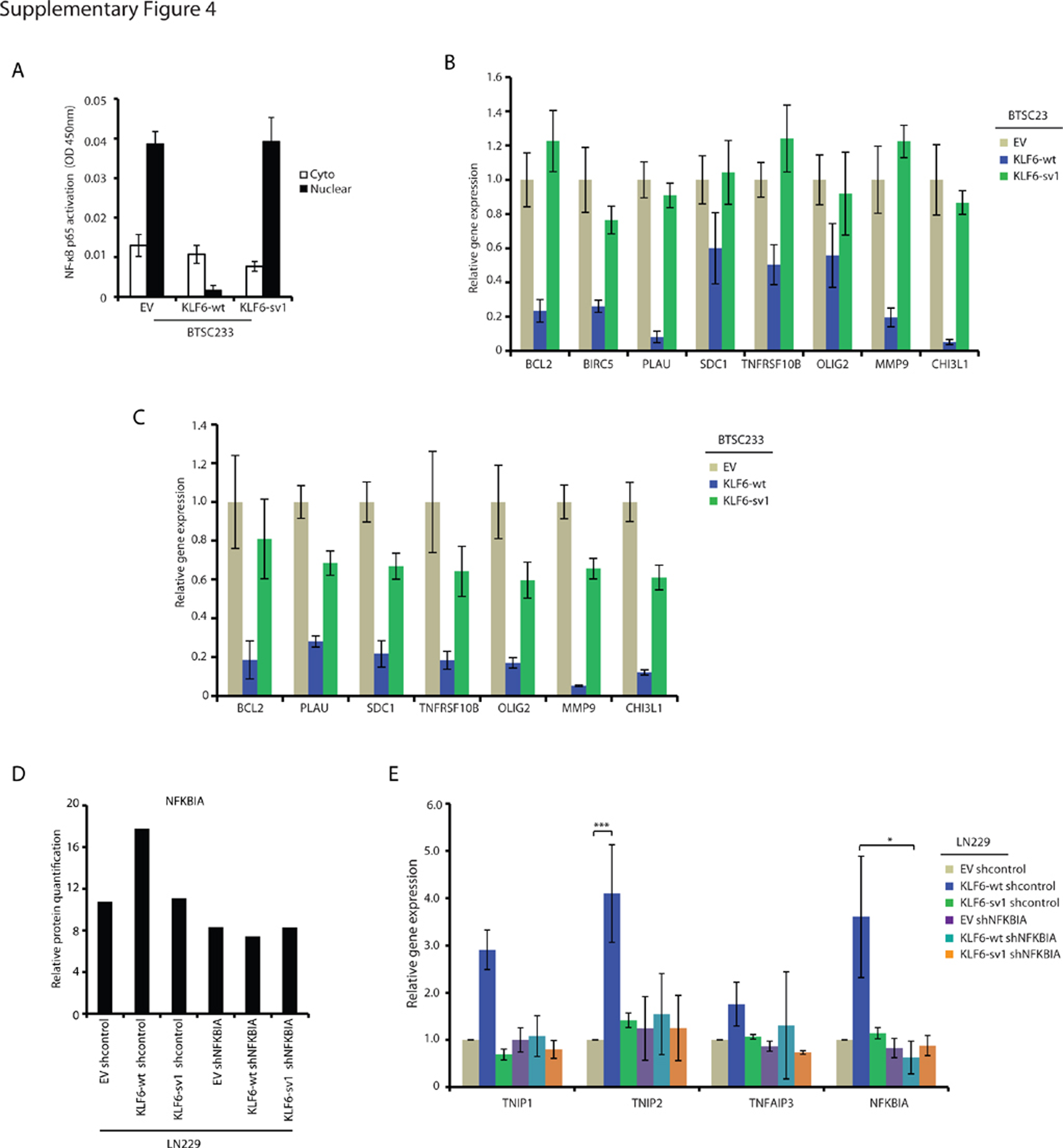

Supplement: Supplementary Figure 4 [file onc2016507x4.tif]

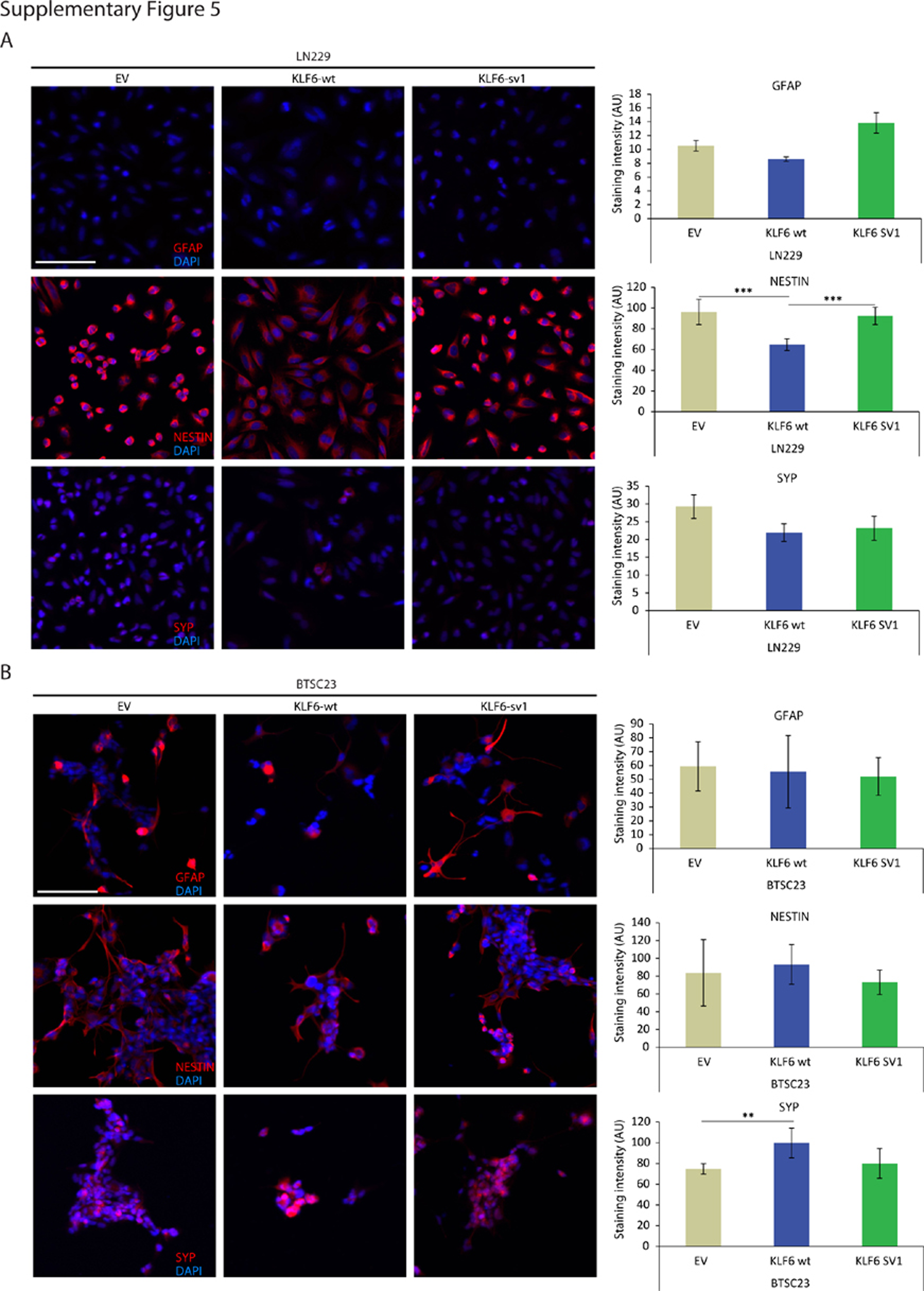

Supplement: Supplementary Figure 5 [file onc2016507x5.tif]

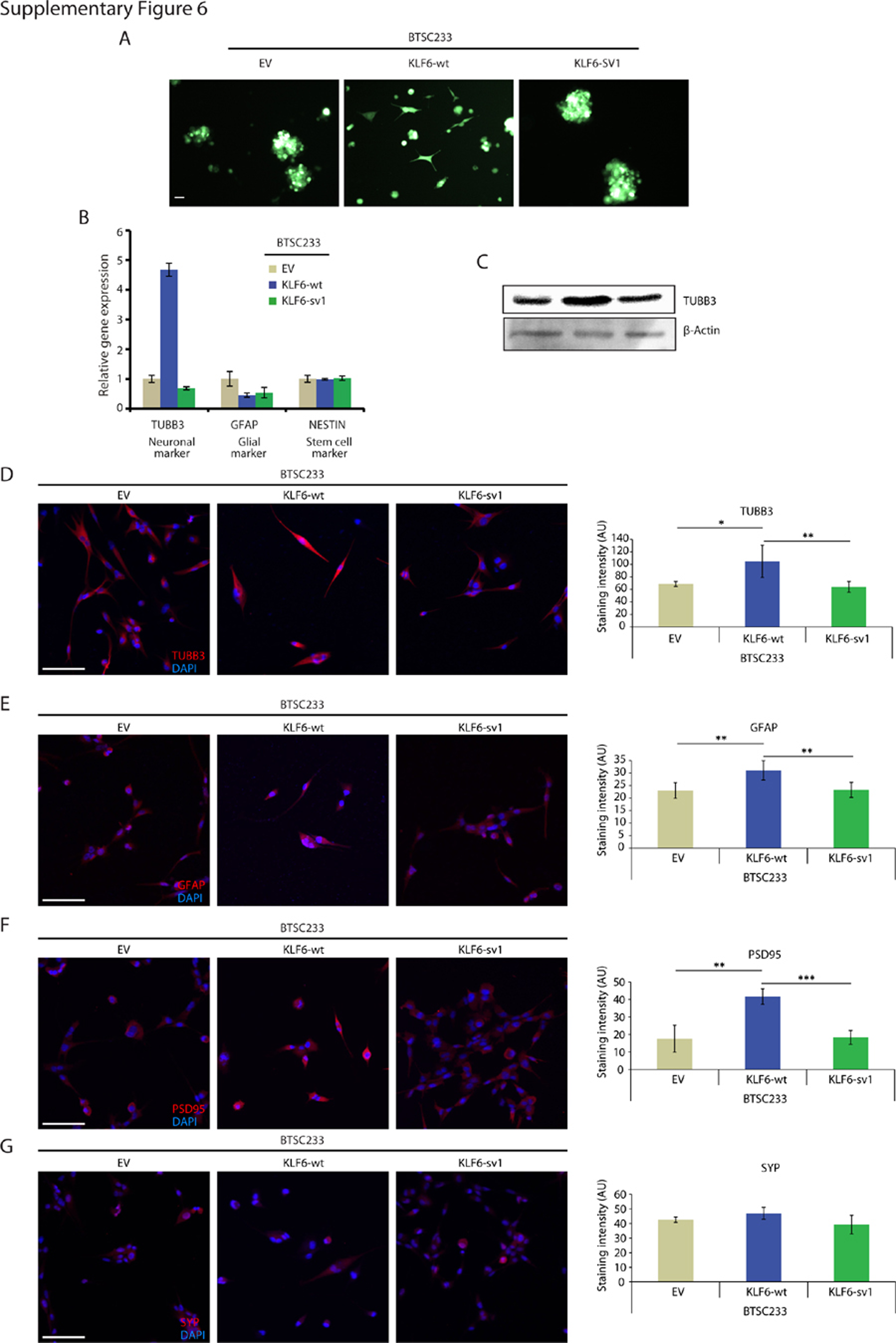

Supplement: Supplementary Figure 6 [file onc2016507x6.tif]

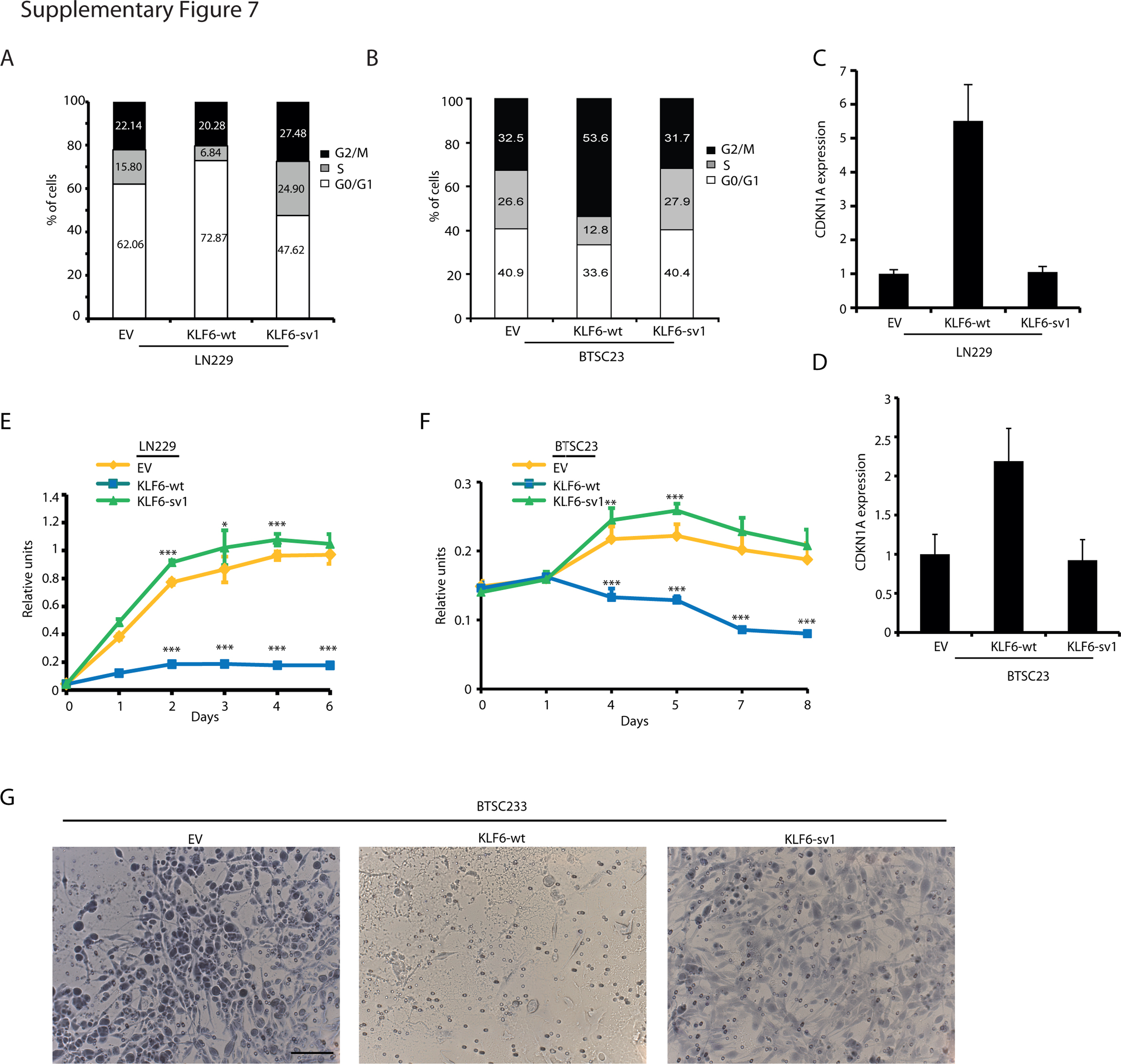

Supplement: Supplementary Figure 7 [file onc2016507x7.tif]

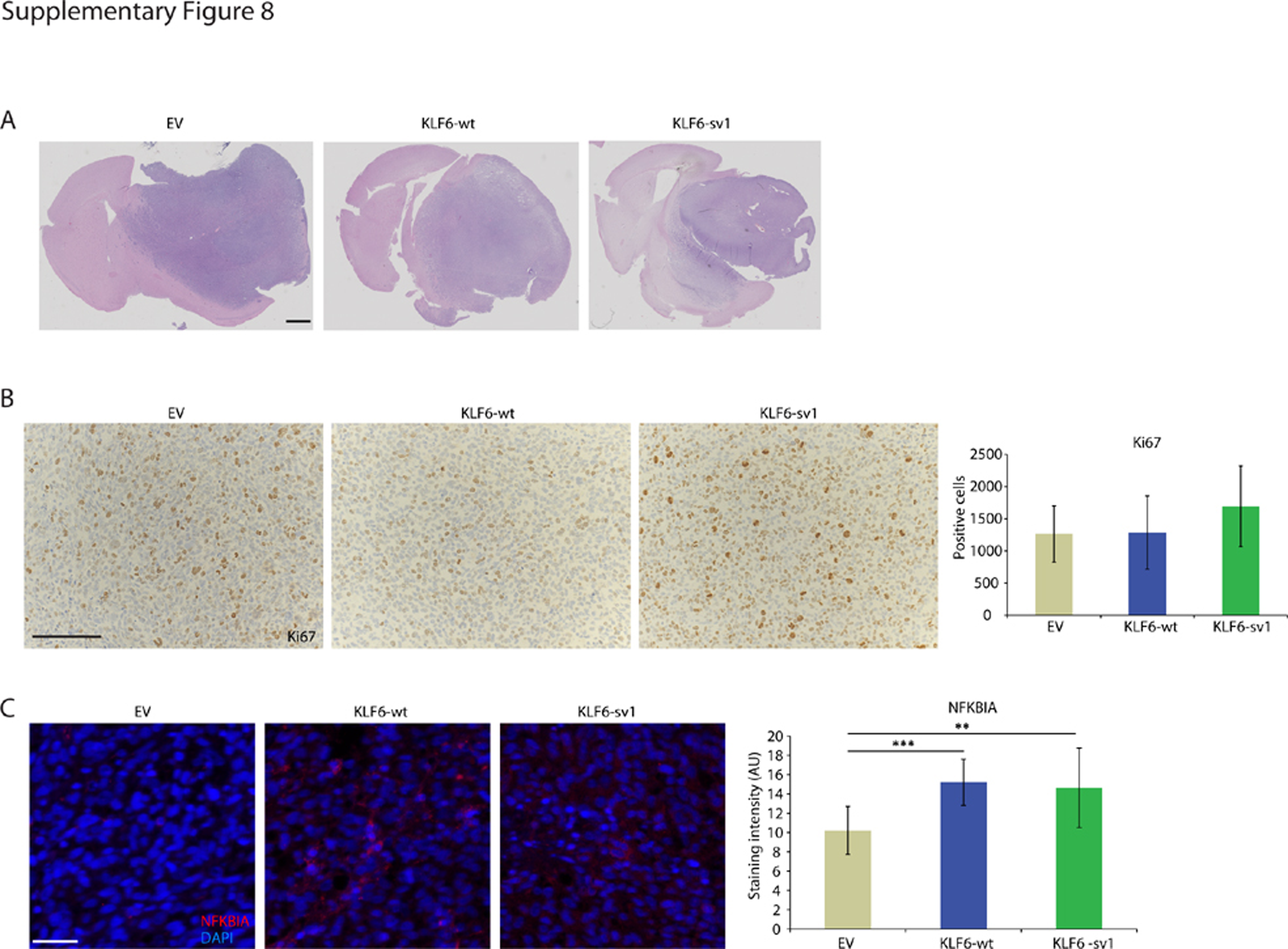

Supplement: Supplementary Figure 8 [file onc2016507x8.tif]
