# Supplementary material for: KLF6 depletion promotes NF-κB signaling in glioblastoma
Source: Oncogene. 2017 Feb 6;36(25):3562–75. doi: 10.1038/onc.2016.507 (PMC5485221; doi:10.1038/onc.2016.507)
Supplement: Supplementary Figure Legends [file onc2016507x9.docx]

**Supplementary Figure Legends**

**Supplementary Figure 1. Gene dosage relationships between NF-kB control genes among each other and with *KLF6* in glioblastoma.** Relationships between gene dosage of four genes (*NFKBIA*, *TNFAIP3*, *TNIP1*, and *TNIP2*) that regulate NF-κB with one another, and with the *KLF6* gene in 175 glioblastomas. Gene dosage values are expressed as ratios of red-to-green fluorescence dye intensity (log_2_R/G). Red lines represent locally weighted least squares (LOWESS) smooth fits. Open squares display the spearman correlation coefficient plus p value for pairwise analyses. Integrating the corresponding correlation coefficients demonstrates that significant p values signify mutually exclusive gene dosage alterations rather than concurrent gene dosage events. This analysis confirms that the co-regulated expression of the NF-κB control genes (and *KLF6*) is not due to concurrent gene copy number variation

**Supplementary Figure 2. Transcription factor binding site analysis of promoters of negative regulators of NF-κB.** Transcription factor binding sites predicted and shared among the promoters of four NF-kB control genes (*TNIP2*, *NFKBIA*, *TNFAIP3*, and *TNIP1*) with their matrix family and transcription factor names. *The p-value represents the probability of obtaining an equal number of sequences with a match in a drawn sample of the same size as the input sequence set.

**Supplementary Figure 3. Characterization of KLF6-wt and KLF6-sv1 transduced cells. (**A-B) qRT-PCR analysis of KLF6-wt and KLF6-sv1 expression in LN229 (A) and BTSC23 (B) cells expressing EV, KLF6-wt, or KLF6-sv1. (C) Immunoblotting analysis for KLF6 and KLF6-sv1 in nuclear and cytoplasmic extracts of LN229 cells expressing EV, KLF6-wt, or KLF6-sv1.

**Supplementary Figure 4. KLF6 inhibits NF-κB signaling in glioblastoma.** (A) Analysis of NF-κB subunit (p65) activation in nuclear and cytoplasmic extracts of BTSC233 cells expressing empty vector EV, KLF6-wt, or KLF6-sv1. Error bars represent mean ± SD. (B-C) qRT-PCR analysis of relative transcript expression of NF-κB targets in BTSC23 (B) and BTSC233 (C) cells expressing EV, KLF6-wt, or KLF6-sv1. (D) Quantification of NFKBIA western blot shown in Figure 4I. (E) qRT-PCR analysis of relative transcript expression of *TNIP1*, *TNIP2*, *NFKBIA*, and *TNFAIP3* in LN229 cells transduced as indicated. *p < 0.05, **p < 0.01, ***p < 0.005. Relative gene expressions are normalized to EV. Representative qRT-PCR of three independent experiments are shown in (B) and (C). Error bars represent mean ± SD (A-C) and relative gene expressions are normalized to EV shcontrol (D).Error bars represent mean ± SD (n=3) (D).

**Supplementary Figure 5. KLF6 reduces stemness marker NESTIN in LN229 and induces expression of neuronal marker SYP in BTSC23.** (A) Immunofluorescence staining, and relative quantification for GFAP, NESTIN, and SYP in LN229 cells expressing EV, KLF6-wt, or KLF6-sv1. Nuclei were stained with DAPI. (B) Immunofluorescence staining, and relative quantification for GFAP, NESTIN, and SYP in BTSC23 cells expressing EV, KLF6-wt, or KLF6-sv1. Nuclei were stained with DAPI. **p<0.01, ***p <0.001. The scale bar represents 50 μm. Error bars represent mean ± SD; n=5.

**Supplementary Figure 6. KLF6 induces expression of differentiation markers in BTSCs.** (A) Microphotographs of GFP-positive BTSC233 cells expressing empty vector (EV), KLF6-wt, or KLF6-sv1, after lentiviral infection. (B) qRT-PCR analysis of neuronal (TUBB3, NEFM), astrocytic (GFAP), and stem cell (NESTIN) markers in BTSC233 cells expressing EV, KLF6-wt, or KLF6-sv1. (C) Immunoblotting of TUBB3 in BTSC23 cells expressing EV, KLF6-wt, or KLF6-sv1. (D-G) Immunofluorescence staining, and relative quantification for TUBB3 (D), GFAP (E), PSD96 (F), and SYP (G) in BTSC233 cells expressing EV, KLF6-wt, or KLF6-sv1. Nuclei were stained with DAPI. *p<0.05, **p<0.01, ***p <0.001. The scale bar represents 50 μm. Error bars represent mean ± SD; n=5.

**Supplementary Figure 7.** (A-B) Cell cycle distribution analysis in LN229 (A) and BTSC23 (B) cells expressing EV, KLF6-wt, or KLF6-sv1. (C-D) qRT-PCR analysis of relative transcript expression of *CDKN1A* in LN229 (C), and BTSC23 (D) cells. (E-F) Analysis of cell viability (MTT assay) in LN229 (E) and BTSC23 (F) cells expressing empty vector (EV), KLF6-wt, or KLF6-sv1. Asterisks indicate statistical significance of KLF6-wt or KLF6-sv1 vs. EV, *p<0.05, **p<0.01, ***p <0.001. (G) Microphotographs of invading BTSC233 cells expressing EV, KLF6-wt, or KLF6-sv1 in a matrigel invasion assay. Relative gene expressions are normalized to EV. Representative qRT-PCR of three independent experiments are shown in (C) and (D). Scale bars represent 100 µm (G). Error bars represent mean ± SD; n=3 (E-F)

**Supplementary Figure 8.** (A) H&E staining of tumors resulting from intracranial injection of LN229 cells expressing EV, KLF6-wt, or KLF6-sv1 into the striatum of NOD/SCID mice. Scale bar represents 1mm. (B) Immunohistochemistry and relative quantification for Ki67 in tumors described in (A). Scale bar represents 100 μm. (C) Immunofluorescence staining and relative quantification for NFKBIA in tumors described in (A). Scale bars represent 20 µm. **p<0.01, ***p<0.001. Error bars represent mean ± SD; n=10 (C); n=5 (KLF6-wt), n=9 (KLF6-sv1), n=4 (EV) (B).
