# Supplementary material for: KLF6 depletion promotes NF-κB signaling in glioblastoma
Source: Oncogene. 2017 Feb 6;36(25):3562–75. doi: 10.1038/onc.2016.507 (PMC5485221; doi:10.1038/onc.2016.507)
Supplement: Supplementary Tables [file onc2016507x10.pdf]

SUPPLEMENTAL TABLES

Supplementary Table 1

Primer sequences used in qRT-PCR analysis

**Primer name**	**Primer sequence (5’ to 3’)**
TNIP1 Sense	GGACCGTACCGGATCTACGA
TNIP1 antiSense	ATTTGAGTCCTTTCCTGTGAGC
TNIP2 Sense	CAGTCGGAACACACAGATGG
TNIP2 antiSense	CCACTTGGCATTGAGGTCTT
TNFAIP3 Sense	TACCCTTGGTGACCCTGAAG
TNFAIP3 antiSense	ATCCAACTTTGCGGCATT
NFKBIA Sense	AACCTGCAGCAGACTCCACT
NFKBIA antiSense	GACACGTGTGGCCATTGTAG
TUBB3 Sense	GCTCAGGGGCCTTTGGACATCTCTT
TUBB3 antiSense	TTTTCACACTCCTTCCGCACCACATC
GFAP Sense	CCGACAGCAGGTCCATGTG
GFAP antiSense	GTTGCTGGACGCCATTGC
NEFM Sense	GTCAAGATGGCTCTGGATATAGAAATC
NEFM antiSense	TACAGTGGCCCAGTGATGCTT
Nestin Sense	AGCCCTGACCACTCCAGTTTAG
Nestin antiSense	CCCTCTATGGCTGTTTCTTTCTCT
BCL2 Sense	CCGCGACTCCTGATTCATT
BCL2 antiSense	TCAGTCTACTTCCTCTGTGATGTTGT
BIRC5 Sense	GTTGCGCTTTCCTTTCTGTCA
BIRC5 antiSense	TCCTTTGCAATTTTGTTCTTGGC
PLAU Sense	GGCAGCACTGTGAAATAGATAAGTC
PLAU antiSense	TCAGATCTGTGGGCATGGTA
SDC1 Sense	CTGCCGCAAATTGTGGCTAC
SDC1 antiSense	TGAGCCGGAGAAGTTGTCAGA
TNFRSF10B Sense	GGAACAACGGGGACAGAAC
TNFRSF10B antiSense	TCAGCTGAGACCAACAGCAG
OLIG2 Sense	CCTAAAGGTGCGGATGCTTA
OLIG2 antiSense	AATCTGGATGCGATTTGAGG
MMP9 Sense	TGTACCGCTATGGTTACACTCG
MMP9 antiSense	GGCAGGGACAGTTGCTTCT

Supplementary Table 2
Primer sequences used in ChIP analysis

**Primer name**	**Primer sequence (5' to 3')**
TNIP1 CHIP Sense	GGCTAAGGCCAGACATGCTA
TNIP1 CHIP antiSense	CTGCCAGTTTTCTTCCTGCT
TNIP2 CHIP Sense	TCGACTCTCCGACAACTCCT
TNIP2 CHIP antiSense	GCCCACCCACAACAGCCCGGC
TNFAIP3 CHIP Sense	GGGGCGAGGGAGTTTCTC
TNFAIP3 CHIP antiSense	ATTTCCACGGGACTTTCCA
NFKBIA CHIP Sense	CAGCAGTTTCCCCATACAGG
NFKBIA CHIP antiSense	AAGGACGCACTGTGGTTAGG
SERPINA1 CHIP Sense	GCCTCCGAGGAAGGCCT
SERPINA1 CHIP antiSense	CTGTCTCTTCTGGCAGGCAC
OLR1 CHIP Sense	ACTGCACCTGGCCAACTTTT
OLR1 CHIP antiSense	TGCAAAGAAAAGAATACACAAAGGA

Supplementary Table 3
Cloning primer sequences

Primer name	Primer sequence (5' to 3')
BstXI-Flag-hKLF6wt Sense	TGGCCACAACCATGGACTACAAGGACGACGATGACAAG GACGTGCTCCCCATGTGCAGCA
PmeI-hKLF6wt antiSense	GCCTTGGTTTAAACTCAGAGGTGCCTCTTCATGTGCAG
BstXI-Flag-hKLF6sv1 Sense	TGGCCACAACCATGGACTACAAGGACGACGATGAC**AAG** GACGTGCTCCCCATGTGCAGCA
PmeI-hKLF6sv1 antisense	GCCTTGGTTTAAACTTAACTCATCACTTCTTGCAAAAC

Supplementary Table 4
Primers for hKLF6 genomic PCR and sequencing

	Primer name	Primer sequence (5' to 3')	Target region	BTs
gPCR1	E1-sense	GATGGAGAGTCTCGGTCACG	Upstream, exon1, intron-exon1 boundaries (606bp_chr10:3827032-3827637)	47
	E1-antisense	GGGTCTGAACCCCAAACAG		
gPCR2	E2-sense	AGGTTTCACCCTCCGACTTT	Exon2, intron-exon2 boundaries (912bp_chr10:3823720-3824631)	50
	E2-antisense	GATTTGTCTGCCCTGACCAC		
gPCR3	E3-sense	CAAGCATTTTGCTGGTCTGA	Exon3, intron-exon3 boundaries (660bp_chr10:3822051-3822710)	46
	E3-antisense	GAAAGGCCAATCTCCAAACA		
gPCR4	E4-sense	ATGAGGCTTGGGAGGAGAAT	Exon3, intron-exon3 boundary and 3'UTR (803bp_ chr10:3821118-3821920)	45
	E4-antisense	CACACAGAAAAGGGGGAGAG		

Supplementary Table 5
Antibodies used in Immunofluorescence and Immunoblotting analysis

Antibody	Catalog No	Source	Company	Purpose
Anti-PSD95	ab18258	rabbit polyclonal	Abcam	IF
Anti-Synaptophysin	ab32127	rabbit monoclonal	Abcam	IF
Anti-Nestin	AB5922	rabbit polyclonal	Millipore	IF
Anti-MMP9	#3852	rabbit polyclonal	Cell Signaling	IF/WB
Anti-GFAP	z0334	rabbit polyclonal	DakoCytomation	IF
Anti-NEFM	sc-161437	goat polyclonal	Santa Cruz Biotechnology	IF
Anti-KLF6 antibody	sc-7158	rabbit polyclonal	Santa Cruz Biotechnology	WB
Anti-alpha Tubulin	ab7291	mouse monoclonal	Abcam	WB
Anti-Flag	F1804	mouse monoclonal	Sigma	WB
Anti-Lamin B	sc-6216	goat polyclonal	Santa Cruz Biotechnology	WB
Anti-Beta Actin	A 2228	mouse monoclonal	Sigma	WB
Anti-IκBα antibody	sc-847	rabbit polyclonal	Santa Cruz Biotechnology	IF/WB
Anti-NFκB p65	sc-8008	mouse monoclonal	Santa Cruz Biotechnology	IF/WB
Anti-βIII-Tubulin	G712A	mouse monoclonal	Promega	IF/WB
Anti-OLIG2	18953	rabbit polyclonal	IBL	WB
Anti-YKL40	4815	rabbit polyclonal	QUIDEL	WB
Anti-Ki67	M7240	rabbit polyclonal	DAKO	IHC
